# Infant Feeding Practices in a Multi-Ethnic Asian Cohort: The GUSTO Study

**DOI:** 10.3390/nu8050293

**Published:** 2016-05-13

**Authors:** Jia Ying Toh, Grace Yip, Wee Meng Han, Doris Fok, Yen-Ling Low, Yung Seng Lee, Salome A. Rebello, Seang-Mei Saw, Kenneth Kwek, Keith M. Godfrey, Yap-Seng Chong, Mary Foong-Fong Chong

**Affiliations:** 1 Singapore Institute for Clinical Sciences, Agency for Science, Technology and Research, Singapore 117609, Singapore; toh_jia_ying@sics.a-star.edu.sg (J.Y.T.); yung_seng_lee@nuhs.edu.sg (Y.S.L.); yap_seng_chong@nuhs.edu.sg (Y.-S.C.); 2 School of Biological Sciences, Nanyang Technological University, Singapore 637551, Singapore; EHYIP1@e.ntu.edu.sg; 3 Department of Nutrition and Dietetics, KK Women’s and Children’s Hospital, Singapore 229899, Singapore; han.wee.meng@kkh.com.sg; 4 Department of Obstetrics & Gynaecology, Yong Loo Lin School of Medicine, National University of Singapore and National University Health System, Singapore 119228, Singapore; obglnld@nus.edu.sg; 5 Saw Swee Hock School of Public Health, National University of Singapore, Singapore 117549, Singapore; yen_ling_low@nuhs.edu.sg (Y.-L.L.); salome_antonette_rebello@nuhs.edu.sg (S.A.R.); seang_mei_saw@nuhs.edu.sg (S.-M.S.); 6 Department of Paediatrics, Yong Loo Lin School of Medicine, National University of Singapore, Singapore 119228, Singapore; 7 Division of Paediatric Endocrinology and Diabetes, Khoo Teck Puat-National University Children’s Medical Institute, National University Health System, Singapore 119074, Singapore; 8 Department of Maternal Fetal Medicine, KK Women’s and Children’s Hospital, Singapore229899, Singapore; Kenneth.Kwek.YC@kkh.com.sg; 9 MRC Lifecourse Epidemiology Unit & NIHR Southampton Biomedical Research Centre, University of Southampton & University Hospital Southampton NHS Foundation Trust, Southampton SO16 6YD, UK; kmg@mrc.soton.ac.uk; 10 Clinical Nutrition Research Centre, Singapore Institute for Clinical Sciences, Agency for Science, Technology and Research, Singapore 117609, Singapore

**Keywords:** infant, feeding practices, Asian, GUSTO

## Abstract

The optimal introduction of complementary foods provides infants with nutritionally balanced diets and establishes healthy eating habits. The documentation of infant feeding practices in multi-ethnic Asian populations is limited. In a Singapore cohort study (GUSTO), 842 mother-infant dyads were interviewed regarding their feeding practices when the infants were aged 9 and 12 months. In the first year, 20.5% of infants were given dietary supplements, while 5.7% took probiotics and 15.7% homeopathic preparations. At age 9 months, 45.8% of infants had seasonings added to their foods, increasing to 56.3% at 12 months. At age 12 months, 32.7% of infants were given blended food, although 92.3% had begun some form of self-feeding. Additionally, 87.4% of infants were fed milk via a bottle, while a third of them had food items added into their bottles. At both time points, more than a third of infants were provided sweetened drinks via the bottle. Infants of Indian ethnicity were more likely to be given dietary supplements, have oil and seasonings added to their foods and consumed sweetened drinks from the bottle (*p* < 0.001). These findings provide a better understanding of variations in infant feeding practices, so that healthcare professionals can offer more targeted and culturally-appropriate advice.

## 1. Introduction

Early life nutrition is fundamental to the development of a child’s full potential [1]. It is well-recognized that the period from birth to two years of age is critical for the promotion of optimal health, growth and behavioral development [2]. Complementary feeding is the introduction of beverages and solid foods other than breast milk or infant formulas to infants. During this transition to table and family foods, it is important that infants are provided with appropriate types and textures of foods and given the opportunity for self-feeding to develop relevant motor skills [3,4]. This is also a time when infants acquire appropriate eating behaviors [5] as early feeding experiences influence food preferences in infancy, and could set the foundation for later food choices and the establishment of life-long habits [6,7]. As infants and toddlers are dependent on parents and other caregivers to feed them, the importance of appropriate infant feeding practices has drawn much interest.

While international and national recommendations and guidelines have been developed to guide parents and caregivers through the complementary feeding process [2,5], the extent to which people follow these recommendations is not well-characterized [8]. Some studies have examined infant supplementation with vitamins, probiotics and homeopathic foods [2,9,10,11], while others have examined the introduction of solids and milk feeding styles [12,13]. In Singapore, infant caregiving practices based on tradition and speculation rather than scientific recommendations is not uncommon [14,15,16]. In addition, socioeconomic status, cultural values and beliefs can influence certain infant feeding practices [12,13]. Current studies have mostly examined infant feeding practices in Caucasian populations [17,18], but less is known about these practices in the Asian context.

This study examined particular aspects of infant feeding practices of mothers and caregivers in the Growing Up in Singapore Towards healthy Outcomes (GUSTO) cohort with a focus on dietary supplements, the addition of oil and seasonings to foods, the provision of blended foods, self-feeding and infant drinking practices. The study evaluated differences in feeding practices among the three major ethnic groups in Singapore, namely the Chinese, Malay and Indian, and focused on the time periods of 9 and 12 months of age.

## 2. Materials and Methods

### 2.1. Study Design and Participants

Data collected from the GUSTO study was used. GUSTO is a prospective birth cohort study started in 2009, which was designed to obtain detailed information about pregnant mothers and their offspring’s health from birth onwards. The study was granted ethical approval by the Institutional Review Board of National University Hospital (NUH) and KK Women’s and Children’s Hospital (KKH).

In brief, 1247 pregnant women were recruited during antenatal clinic visits (<14 weeks gestation) at NUH and KKH from June 2009 to September 2010. The participants recruited had to be of Chinese, Malay or Indian ethnicity, with homogenous parental ethnic background, aged 18–50, without significant health conditions such as type I diabetes mellitus and psychosis, with the intention to deliver in NUH or KKH and to reside in Singapore for the next five years, along with the willingness to donate cord, cord blood and placenta. Written informed consent was obtained from all of the participants. More details of the study have been previously described in Soh *et al*. (2011) [19]. For the present study, only women with singleton pregnancies who attended all three (6th, 9th and 12th months) postnatal clinic visits were included. A total of 842 mother-infant dyads were examined. (Figure S1).

### 2.2. Assessment of Maternal and Infant Characteristics

Data on maternal ethnicity, age, body mass index (BMI), education level, monthly household income, employment status, alcohol consumption and smoking before pregnancy, parity and dietary intake were assessed during recruitment and at the 26–28 weeks antenatal clinic visit. Missing information for BMI (*n* = 25), education level (*n* = 7), monthly household income (*n* = 54), employment status (*n* = 16), alcohol consumption (*n* = 12), smoking (*n* = 10) and parity (*n* = 8) were imputed with their respective median values for statistical analysis.

Infant data on birth weight, length, gestational age, and gender were collected at birth. Infants born <37 gestational weeks were defined as pre-term while infants with birth weight <10th percentile of the global reference [20] were considered small for gestational age (SGA). Breastfeeding status was determined from the type of infant feeding (exclusive *vs*. partial *vs*. formula only) starting from three weeks to 12 months of age. Information concerning the main food decision-maker, age of introduction of first foods were collected retrospectively at nine months. Missing information for birth length (*n* = 2) and age of introduction of first foods (*n* = 4) were imputed with their respective median values for statistical analysis.

### 2.3. Assessment of Feeding Practices

Data on infant feeding practices was collected from the mothers or caregivers through interviewer-administered questionnaires at the 9th and 12th month postnatal clinic visits. The questions assessed the following aspects of infant feeding practices: (1) Use of dietary supplements; (2) Addition of oil and seasonings to foods (such as salt and soya sauce, *etc*.); (3) Use of blended foods and self-feeding methods and; (4) Drinking practices for milk and sweetened beverages.

### 2.4. Statistical Analysis

All data collected were analyzed using SPSS Version 19.0 (IBM Corp, New York, NY, USA) for Windows. Pearson’s chi-square test of independence was conducted to assess maternal socio-demographic characteristics, infant characteristics and feeding practices among and between ethnic groups and across time points. A two-sided test of equality for column proportions with Bonferroni correction was used to identify specific differences between ethnic groups. For two by two tables and data with values less than five counts, Yate’s continuity correction and Fisher’s Exact test were used to assess significance, respectively, instead of the chi-square test. Results with *p* values ≤ 0.05 were considered significant.

## 3. Results

### 3.1. Characteristics of Participants

The characteristics of mothers and infants in this study, stratified by ethnic groups, are presented in Table 1a,b. The ethnic distribution was 60.6% Chinese, 23.0% Malay and 16.4% Indian. The median age range of the mothers was 30–34 years old, with Chinese mothers on average being generally older than Indian and Malay mothers. The majority of mothers had post-secondary or higher education (72.4%), were employed (71.0%), were non-smokers (88.4%) and did not consume alcohol before pregnancy (65.6%). About half of the mothers in this study had mid-range household income (56.5%), and had one or more children prior to participation in this study (55.3%).

For infants, mean gestational age was 38.3 ± 1.4 weeks (*n* = 57; 6.8% born pre-term), while mean birth weight and length were 3.12 ± 0.4 kg and 48.7 ± 2.3 cm, respectively. A minority of infants were born small for gestational age (SGA) (*n* = 104; 12.4%). The majority of infants (86.4%) were breastfed between birth and 12 months of age. More than half of the infants were weaned between six and seven months (58.9%). The most common first foods given were rice cereal (53.7%), followed by non-rice cereal (17.2%) and rice porridge (12.1%). At nine months, mothers were generally the main food decision maker (70.8%), while grandparents and other secondary caregivers made up 17.1% and 12.1% respectively. Similar numbers were observed at 12 months.

### 3.2. Infant Feeding Practices

#### 3.2.1. Use of Dietary Supplements

The number and percentage of infants who were given dietary supplements in their first year are presented in Table 2.

#### 3.2.2. Vitamin and Mineral Supplements

In the first year of life, 20.5% of infants were given vitamin and mineral supplements. The three most common types taken were multivitamins (9.0%), minerals such as iron and calcium (8.4%) and fish oil (8.2%); others included individual vitamins (4.0%) (e.g., vitamin A, B12, C and D). Other types of dietary supplements consumed included protein powder, spirulina, goat’s milk tablets, plant source oils and lysine supplements (2.0%).

At 9 and 12 months of age, a greater percentage of Indian infants (15.9% and 19.6%) were taking vitamin or mineral supplements compared to Chinese (11.8% and 16.5%) and Malay (10.3% and 13.9%) infants, although these ethnic differences were not statistically significant (*p* > 0.20 for both time points) (Table 2).

#### 3.2.3. Probiotic Supplements

A small percentage (5.7%) of infants was either given probiotic supplements such as probiotics drops or powder (4.4%) or probiotics-enriched foods (1.3%) such as cultured milk and cereals fortified with probiotics in their first year of life. Probiotics were provided more commonly to infants were between 4–7 months (2.4%) and 8–12 months (2.9%), and to a small proportion in the 0–3 months group (0.5%). At 9 and 12 months of age, the percentage of Chinese infants (4.1% and 4.7%) taking probiotics was significantly higher than Malay (0.5% at both time points) and Indian (0.7% and 1.4%) infants (*p* ≤ 0.01 for both time points) (Table 2).

#### 3.2.4. Homeopathic Supplements

More than a tenth (15.7%) of infants were introduced to homeopathic supplements within the first 12 months of age. Some of the homeopathic supplements that were given to the infants included traditional Chinese medicine, Indian herbs and Gripe Water. Most infants were provided homeopathic supplements on an *ad hoc* basis (9.3%), while some were given on a daily basis (6.1%) and a minority had on alternate days (0.6%). It was reported that these homeopathic supplements were recommended by family and friends (10.0%), self (4.2%), healthcare professionals (1.8%) or through sales (0.2%).

Overall, of 842 mother-child dyads in this study, 35.5% of the infants were given at least one type of dietary supplement during their first year of life. Mothers of these infants tended to be older (aged 30–34 years old), and had post-secondary education compared to those whose infants were not given supplements (*p* < 0.001 for both). Supplementation was also provided by a small number of mothers who were on special diets during pregnancy (diabetic diet 1.0%, low fat diet 2.0%), although the differences were not statistically significant (*p* = 0.946). Additionally, infants who were given dietary supplements had lower birth weight (3.08 ± 0.5 kg; *p* = 0.105), shorter birth length (48.5 ± 2.6 cm; *p* = 0.258) and gestational age (38.2 ± 1.8 weeks; *p* = 0.069) than those not supplemented (Table S1a,b).

#### 3.2.5. Addition of Oil and Seasonings to Foods

The percentage of infants who had oil added to their foods increased from 23.8% to 32.7% at 9 months to 12 months of age. At both time points, Indian infants (42.0% and 52.9%) were significantly more likely to have oil added to their foods than Chinese (20.2% and 28.2%) and Malay (20.1% and 29.9%) infants (*p* < 0.001 for both time points) (Table 3).

For seasonings, 45.8% of infants had seasonings added to their foods at 9 months of age and this percentage increased to 56.3% when the infants were 12 months of age. At nine months of age, Indian infants (64.5%) were significantly more likely to have seasonings added to their foods than Chinese (39.6%) and Malay (49.0%) infants (*p* < 0.001). This pattern persisted at 12 months of age (51% Chinese, 59.8% Malay, 71.0% Indian; *p* < 0.001) (Table 3).

A subset analysis of pre-term infants showed that they were more likely to have oil added to their food as compared to infants born of term at 12 months of age (45.6% pre-term, 31.7% term; *p* = 0.044). Additionally, more SGA infants (55.8%) had seasoning added to their food as compared to appropriate for gestational age (AGA) infants (44.4%; *p* = 0.039) at nine months of age. Infants that were not born as the first child also had a higher likelihood of being introduced to oil at 9 months of age (27.3% not first child, 19.4% first child; *p* = 0.010) and seasoning at both 9 and 12 months of age (51.1%, 60.5% not first child, 39.4%, 51.1% first child at 9 and 12 months, respectively; *p* < 0.01 for all).

#### 3.2.6. Use of Blended Foods and Self-Feeding Methods

At nine months of age, half of the infants (49.9%) were still provided blended food. The reasons cited were ease of feeding due to lack of teeth or infants being unable to manage unblended food. By 12 months of age, the percentage of infants given blended food decreased to a third of the cohort (32.7%) with similar reasons cited. At both time points, a significantly greater proportion of Indian infants (62.3% and 40.6%) were given blended food as compared to Chinese (45.5% and 32.7%) and Malay (52.6% and 26.8%) infants (*p* < 0.05 for both time points) (Table 3).

At nine months of age, 78.6% of the infants had started some form of self-feeding. Among these infants, the majority of them took to finger foods (77.4%), while a fifth of them could feed themselves using a cup (19.6%). Only a small proportion could feed themselves using a spoon (7.2%). By 12 months of age, the percentage of self-feeding infants increased to 92.3%, with the majority taking to finger foods (91.7%), a third to cup-feeding (33.8%) and an increase in the number of infants who could manage spoon-feeding (13.7%). The most common ages for initiation of finger food feeding, cup-feeding and spoon-feeding among the infants were 9, 11 and 11 months old, respectively. At 9 and 12 months of age, the percentage of Malay infants that were self-feeding (89.7% and 96.4%) was significantly higher than Chinese (73.1% and 90.6%) and Indian (83.3% and 92.8%) infants (*p* < 0.05 both time points) (Table 3).

In addition, infants who were not born as the first child practiced self-feeding more as compared to those who were the only child at 12 months of age (94.4% not first child, 89.6% first child; *p* = 0.014).

### 3.3. Infant Drinking Practices

Table 4 shows the data on drinking practices of milk and sweetened beverages among the infants.

#### 3.3.1. Milk Feeding Practices

At nine months of age, the majority of infants were still consuming milk via a bottle (85.6%), fewer were directly breastfed (13.1%) and a minority fed via other methods such as a cup, spoon or straw (1.3%). At 12 months of age, bottle-feeding remained the most common method of feeding milk (87.4%), with the percentage of those directly breastfed decreasing slightly (10.7%). At both 9 and 12 months of age, fewer Malay infants (10.8% and 7.2%) were breastfed compared to Chinese (12.5% and 11.4%) and Indian (18.1% and 13.0%) infants. These differences were statistically significant at 9 months of age (*p* = 0.01), but not at 12 months of age (*p* = 0.153) (Table 4).

Furthermore, subset analysis in relation to parity showed that infants who were not born as the first child were more likely to be breastfed as compared to infants who were the only child at both 9 and 12 months of age (15.5%, 13.3% not first child, 10.1%, 7.4% first child at 9 and 12 months, respectively; *p* < 0.05 for all).

#### 3.3.2. Addition of Food Items into Milk Bottle

At nine months of age, slightly more than 30% of infants had food items added into their milk bottle when feeding. This proportion remained similar at 12 months of age. The most common food items added were pureed food, cereal and rusks. Other food items included extra milk powder or water, sweetener (honey or sugar), supplements and other beverages. At both time points, more Indian infants (38.4% and 39.9%) had food items added into their milk bottle as compared to Chinese (31.0% and 33.7%) and Malay (27.8% and 29.4%) infants. These differences were, however, not statistically significant (*p* > 0.10 for both time points). Interestingly, infants who had sweetener added into their milk bottle during feeding were mostly of Indian ethnicity, while those who had extra water or milk powder added were mostly Chinese (Table 4).

Pre-term infants were also more likely to have food items added into their milk bottle as compared to term infants at both 9 and 12 months of age (9 months of age: 49.1% pre-term, 30.2% term; *p* = 0.005; and 12 months of age: 50.9% pre-term, 32.5% term; *p* = 0.007).

#### 3.3.3. Juices and Sweetened Drinks

At nine months of age, 31.6% of the infants were not provided juices and sweetened drinks. The remaining 39.7% of them drank juices and sweetened drinks from a milk bottle, while 28.7% used other methods like a cup or spoon. The trend was similar at 12 months of age, with a slight drop in the percentage of infants drinking juices and sweetened drinks from milk bottles (34.2%). Among the ethnic groups at 9 and 12 months of age, the percentage of Chinese infants fed juices and sweetened drinks (62.9% and 67.8%) was significantly lower than Malay (74.7% and 82%) and Indian (79.7% and 82.6%) infants (*p* < 0.001 for both time points) (Table 4).

At 9 and 12 months of age, using milk bottle to drink juices and sweetened drinks appeared to be most common among Indian infants (49.3% and 44.2%), while cup usage was popular among Malay infants (18.0% and 29.9%) (Table 4).

#### 3.3.4. Water

At nine months of age, the use of a milk bottle was the most common way of drinking water by the infants (67.8%), followed by the use of a cup (17.3%). This trend persisted at 12 months of age, with a slight decrease in bottle usage (54.5%) and an increase in cup usage (23.0%). At both 9 and 12 months of age, cup usage was significantly less common among Chinese infants (14.3% and 17.6%), as compared to Malay (23.3% and 30.9%) and Indian (20.3% and 31.9%) infants (*p* < 0.05 for both time points) (Table 4).

### 3.4. Source of Advice

About half of the mothers (56.3%) reported to seek advice on infant feeding. A majority of them (60%) reported obtaining advice from family and friends, while a third (35%) from the media and the rest (17.1%) from health professionals. These were related to aspects of food choice, nutrition and weaning of infants onto solids (36.9%, 34.4% and 35.9%, respectively).

## 4. Discussion

While several local studies have examined breastfeeding [16,21,22,23], picky eating [24] and parenting practices [14], little research has been done on infant feeding practices in Asian populations. To our knowledge, this study is the first to provide data on infant feeding practices in a multi-ethnic cohort. Here, we observed variations in infant feeding practices at 9 and 12 months of age, and also differences among ethnic groups.

### 4.1. Use of Dietary Supplements

In our study, about a fifth of the infants were provided with vitamin and mineral supplementation. This is in line with other studies such as the Iowa Fluoride study, where 31.7% of infants were given supplements at 24 months of age [10], in the Feeding Infants and Toddlers study in United States, where 19% of 6- to 11-month-olds and 31% of 12- to 24-month-olds were given supplements [25], and in the Taiwan Birth Cohort Study, where 37% of infants were given multivitamins by 6 months of age [26].

Vitamin and mineral supplements are generally not recommended as most healthy infants should be able to obtain adequate nutrients from a combination of breast milk, commercially available fortified formula milk and fortified complementary foods. The exceptions are for infants fed a plant-based diet, those persistently under-eating [2,10,27,28] or are exclusively breastfed with limited sun exposure or access to fortified complementary foods [9]. Excessive supplementation of certain nutrients may also potentially lead to adverse effects [29].

Even though vitamin D supplementation of 400 IU per day for infants and children is strongly encouraged in Western countries [30], this is not routinely provided for infants and children in Singapore. Geographically located near the equator (1°N), sunlight is available all year round in Singapore and it is regarded that regular exposure of 10–15 min would produce sufficient vitamin D, reducing the need for supplementation in our infants [31]. In our study, a minority of mothers (2.1%) were vegetarians, of which mostly were from the Indian ethnic group. It is likely that their infants were also following a plant-based diet, thus explaining the higher prevalence of supplementation among Indian infants. The majority of the infants who were provided with dietary supplementation were partially breastfed, and half of them were taking fortified rice cereals. It is possible that some mothers provide dietary supplements to their infants out of concern for their smaller birth size or shorter gestational age. Whether some of these infants actually require dietary supplementation is questionable and warrants further investigation.

The reported prevalence of homeopathic supplementation in our study (15.7%) was slightly higher than the prevalence of 9% reported by the Infant Feeding Practices Study II in the United States [11]. We believe this could be due to cultural beliefs whereby traditional systems of medicine such as Traditional Chinese Medicine, Ayuverdic and Jamu are still followed and adopted as alternative supplementation for infant health [15].

The provision of probiotics may be concomitant with affordability and this may explain the higher intakes amongst Chinese infants, as more Chinese mothers fell within the highest monthly household income tertile.

### 4.2. Addition of Oil and Seasonings to Foods

An increasing trend of adding oil and seasonings during the preparation of infant’s food from 9 to 12 months of age was observed in our cohort. A possible reason could be the introduction of more table foods at the age of 12 months [32]. According to World Health Organization (WHO) guidelines, up to 5 g/day of addition fat is required in the diet if animal-source food containing some fat is consumed by the infant on a regular basis (e.g., whole cow’s milk) [33]. In addition, an energy contribution of 30%–40% by total fat intake is considered reasonable [34]. In our study, the infants were either consuming breast milk or formula milk at 9 and 12 months of age and these should provide sufficient sources of fat. The addition of fat to complementary foods could potentially affect the overall energy density, and excessive intake could potentially increase the likelihood of childhood obesity, although the evidence for this is still limited [35,36].

Some studies suggest that mothers add salt to their child’s food to mask the bitterness of certain foods such as vegetables, so that consumption is enhanced [37,38]. However, early exposure to salt is of concern as it can increase later preferences for salty food [39]. High sodium intake during the first six months of life has also shown to be associated with higher blood pressure in infancy and potentially long-term negative health consequences such as hypertension and cardiovascular disease in adulthood [40,41].

### 4.3. Use of Blended Foods and Self-Feeding Methods

Providing textured foods to infants is important for them to learn how to chew. WHO and local guidelines recommend that finely minced/chopped foods should be provided at 10 to 12 months of age. Our data showed that a third of the cohort was still provided with blended food at 12 months. This is of concern as there is suggestive evidence that delaying the introduction of “lumpy” solid foods beyond 10 months of age can increase the risk of feeding difficulties later on [17]. Unexpectedly, most of the infants were reported to be able to self-feed using finger foods by 12 months of age, suggesting that infants were able to consume textured foods but still offered blended foods. There is evidence to show that some mothers tend to provide pureed food as they are ingested more efficiently and require less time for feeding [42]. In our study, Malay infants appear to be more independent in feeding as compared to Chinese and Indian infants, and this could be attributed to the Malay cultural practice of eating using their hands [43].

### 4.4. Drinking Practices for Milk and Sweetened Beverages

The usage of the milk bottle remained the most common method for drinking milk at both 9 and 12 months among the infants. Bottle-fed infants have been shown to consume significantly more milk, and hence consume more protein than cup-fed infants [44,45]. Studies have shown that prolonged bottle-feeding, beyond the weaning age of 12 to 14 months, increases the risk of being in a higher BMI category by 3% for each additional month delayed [46,47,48]. This may be in part due to the relative inability of an infant to self-regulate energy intake when he/she is fed from the bottle [49]. This remains to be monitored in our infants as they grow beyond the age of one year.

Similarly, a significant proportion of infants in the study drank juice and sweetened drinks from a milk bottle at both 9 and 12 months of age while a third of them had food items or extra milk powder added into their milk bottle when feeding. These trends appeared to be most common among Indian infants. Even though fruit juices contain some vitamins, they also contain fructose and other sugars, which taken in excess via the milk bottle can increase the risk of dental caries and tooth erosion [34,50,51]. Sugar alcohols found in juices and sweetened drinks could also cause non-specific chronic diarrhea in infants [34]. Amongst these, there is also the effect on the development of taste preference for sweet foods with early exposure [52]. By 12 months of age, infants are encouraged to drink from cups instead [2].

Studies have suggested that the belief behind the practice of adding food items into milk bottle is to increase satiation, soothe the infant and encourage the child to sleep better at night [53,54,55]. The above practices may lead to excessive intakes and the overconsumption of calories, displacing other nutrient-rich foods in the diet.

## 5. Study Strengths and Limitations

This study examined aspects of feeding practices which have not been previously studied in Asian populations. The response rate of the study was relatively high, with at least 90% responses for the various questions. However, it should be acknowledged that data obtained from the questionnaires were all self-reported and some of the questions were retrospectively asked. As such, there might be recall bias leading to over-representation or under-representation of some of the data. The reasons behind the various feeding practices were not captured in the GUSTO study and should be examined for future work.

## 6. Conclusions

Trends in infant feeding practices at 9 and 12 months of age were observed in a multi-ethnic Asian population and differences were noted among the three ethnic groups. Feeding practices such as the early introduction of oil and seasonings, the addition of extra food items into the bottle and the provision of sweetened beverages may potentially contribute to adverse health outcomes later in life. An understanding of existing local infant feeding practices can better inform public health policymakers on developing locally appropriate feeding recommendations based on guiding principles and standards set by WHO. These guidelines should be actively promoted through the media and educational talks or materials. An avenue could also be established for caregivers to obtain proper advice and support. Health professionals can also provide more targeted and culturally appropriate health advice to cater to differing feeding beliefs among the ethnic groups. Our data also serves as a platform for future work to ascertain if feeding practices like these have an association with later metabolic risk.

## Figures and Tables

**Table 1a:** Profile of the mothers in the study (*n* (%)).

	Total Study Population	Chinese	Malay	Indian	*p*-Value
	(*n* = 842)	(*n* = 510)	(*n* = 194)	(*n* = 138)	
**Age (years)**					<0.001
18–24	79 (9.4)	22 (4.3)	38 (19.6)	19 (13.8)	
25–29	245 (29.1)	127 (24.9)	82 (42.3)	36 (26.1)	
30–34	291 (34.6)	196 (38.4)	37 (19.1)	58 (42.0)	
≥35	227 (27.0)	165 (32.4)	37 (19.1)	25 (18.1)	
**BMI (26 weeks)**					<0.001
≤18.5–22.9	188 (22.3)	142 (27.8)	27 (13.9)	19 (13.8)	
23.0–27.5	406 (48.2)	277 (54.3)	69 (35.6)	60 (43.5)	
>27.5	248 (29.5)	91 (17.8)	98 (50.5)	59 (42.8)	
**Highest education**					<0.001
Secondary or lower	232 (27.6)	119 (23.3)	87 (44.8)	26 (18.8)	
Post-secondary	306 (36.3)	173 (33.9)	94 (48.5)	39 (28.3)	
University or higher	304 (36.1)	218 (42.7)	13 (6.7)	73 (52.9)	
**Monthly household income (S$)**					<0.001
≤1999	114 (13.5)	48 (9.4)	46 (23.7)	20 (14.5)	
2000–5999	476 (56.5)	256 (50.2)	138 (71.1)	82 (59.4)	
≥6000	252 (29.9)	206 (40.4)	10 (5.2)	36 (26.1)	
**Employment status**					<0.001
Employed	598 (71.0)	127 (24.9)	56 (28.9)	61 (44.2)	
Unemployed	244 (29.0)	383 (75.1)	138 (71.1)	77 (55.8)	
**Alcohol consumption before pregnancy**					<0.001
Yes	290 (34.4)	241 (47.3)	25 (12.9)	24 (17.4)	
No	552 (65.6)	269 (52.7)	169 (87.1)	114 (82.6)	
**Smoking before pregnancy**					<0.001
Yes	98 (11.6)	39 (7.6)	49 (25.3)	10 (7.2)	
No	744 (88.4)	471 (92.4)	145 (74.7)	128 (92.8)	
**Parity**					0.011
No child	376 (44.7)	247 (48.4)	81 (41.8)	48 (34.8)	
≥1 child	466 (55.3)	263 (51.6)	113 (58.2)	90 (65.2)	

Missing information for BMI (*n* = 25), education level (*n* = 7), monthly household income (*n* = 54), employment status (*n* = 16), alcohol consumption (*n* = 12), smoking (*n* = 10) and parity (*n* = 8) were imputed with their respective median values. *p*-value indicates significance level among three ethnic groups (Chinese, Malay, Indian); *p* ≤ 0.05 is considered significant.

**Table 1b:** Profile of the infants in the study.

	Total Study Population	Chinese	Malay	Indian	*p*-Value
	(*n* = 842)	(*n* = 510)	(*n* = 194)	(*n* = 138)	
**Birth weight (kg) (mean (SD))**	3.12 (0.4)	3.13 (0.4)	3.15 (0.4)	3.02 (0.5)	0.016
**Birth length (cm) (mean (SD))**	48.7 (2.3)	48.8 (2.3)	48.4 (2.1)	48.5 (2.5)	0.122
**Gestational age (weeks) (mean (SD))**	38.3 (1.4)	38.4 (1.4)	38.1 (1.3)	38.3 (1.8)	0.038
**Gender (*n* (%))**					0.569
Male	433 (51.4)	261 (51.2)	105 (54.1)	67 (48.6)	
Female	409 (48.6)	249 (48.8)	89 (45.9)	71 (51.4)	
**Breastfeeding (BF) status (*n* (%))**					<0.001
Exclusively BF first three months	164 (19.5)	116 (22.7)	17 (8.8)	31 (22.5)	
Partial BF	563 (66.9)	330 (64.7)	142 (73.2)	91 (65.9)	
Never BF	115 (13.7)	64 (12.5)	35 (18.0)	16 (11.6)	
**Main food decision maker (*n* (%))**					0.018
Mother	596 (70.8)	339 (66.5)	149 (76.8)	108 (78.3)	
Grandparent	144 (17.1)	101 (19.8)	25 (12.9)	18 (13.0)	
Others ^1^	102 (12.1)	70 (13.7)	20 (10.3)	12 (8.7)	
**Age of introduction of first foods (weeks) (*n* (%))**					<0.001
≤15	20 (2.4)	4 (0.8)	10 (5.2)	6 (4.3)	
16–23	292 (34.7)	162 (31.8)	83 (42.8)	47 (34.1)	
24–31	496 (58.9)	325 (63.7)	90 (46.4)	81 (58.7)	
≥32	34 (4.0)	19 (3.7)	11 (5.7)	4 (2.9)	
**First foods (*n* (%))**					0.002
Rice cereal ^2^	452 (53.7)	278 (54.5)	116 (59.8)	58 (42.0)	
Non-rice cereal ^3^	145 (17.2)	99 (19.4)	23 (11.9)	23 (16.7)	
Rice porridge ^4^	102 (12.1)	65 (12.7)	19 (9.8)	18 (13.0)	
Fruit puree	59 (7.0)	29 (5.7)	14 (7.2)	16 (11.6)	
Vegetable puree	31 (3.7)	15 (2.9)	8 (4.1)	8 (5.8)	
Rice	19 (2.3)	9 (1.8)	4 (2.1)	6 (4.3)	
Baby biscuit	15 (1.8)	4 (0.8)	8 (4.1)	3 (2.2)	
Others	14 (1.7)	8 (1.6)	1 (0.5)	5 (3.6)	
Not answered	5 (0.6)	3 (0.6)	1 (0.5)	1 (0.7)	

Missing information for birth length (*n* = 2) and age of introduction of first foods (*n* = 4) was imputed with its median value. *p*-value indicates significance level among three ethnic groups (Chinese, Malay, Indian); *p* ≤ 0.05 is considered significant. ^1^ Others consist of father (*n* = 7), secondary caregiver (*n* = 44), shared responsibility (*n* = 43), not reported (*n* = 8); ^2^ Iron-fortified rice cereal (*n* = 394), non-iron fortified rice cereal (*n* = 58); ^3^ For example, of non-rice cereal are oats, wholegrain and fruit cereals. ^4^ Plain rice porridge (*n* = 85), flavored rice porridge (*n* = 17).

**Table 2:** Comparison of dietary supplementation and consumption of probiotics at 9 and 12 months among Chinese, Malay and Indian infants (*n* (%)).

	Total Infants at 9 Months	Chinese	Malay	Indian	*p*-Value	Total infants at 12 Months	Chinese	Malay	Indian	*p*-Value
	(*n* = 842)	(*n* = 510)	(*n* = 194)	(*n* = 138)		(*n* = 842)	(*n* = 510)	(*n* = 194)	(*n* = 138)	
**Consumed vitamin or mineral supplements**					0.279					0.390
Yes	102 (12.1)	60 (11.8)	20 (10.3)	22 (15.9)		138 (16.4)	84 (16.5)	27 (13.9)	27 (19.6)	
No	740 (87.9)	450 (88.2)	174 (89.7)	116 (84.1)		704 (83.6)	426 (83.5)	167 (86.1)	111 (80.4)	
**Consumed probiotics**					0.010					0.005
Yes	23 (2.7)	21 (4.1) ^a^	1 (0.5) ^b^	1 (0.7) ^a,b^		27 (3.2)	24 (4.7) ^a^	1 (0.5) ^b^	2 (1.4) ^a,b^	
No	819 (97.3)	498 (95.9) ^a^	193 (99.5) ^b^	137 (99.3) ^a,b^		815 (96.8)	486 (95.3) ^a^	193 (99.5) ^b^	136 (98.6) ^a,b^	

*p*-value indicates significance level among three ethnic groups (Chinese, Malay, Indian); *p* ≤ 0.05 is considered significant. ^a,b^ Values in the same row not sharing the same subscript are significantly different at *p* ≤ 0.05 in the two-sided test of equality for column proportions.

**Table 3:** Comparison of feeding practices at 9 and 12 months among Chinese, Malay and Indian infants (*n* (%)).

	Total Infants at 9 Months	Chinese	Malay	Indian	*p*-Value	Total Infants at 12 Months	Chinese	Malay	Indian	*p*-Value
	(*n* = 842)	(*n* = 510)	(*n* = 194)	(*n* = 138)		(*n* = 842)	(*n* = 510)	(*n* = 194)	(*n* = 138)	
**Addition of oil**					<0.001					<0.001
Yes	200 (23.8)	103 (20.2) ^a^	39 (20.1) ^a^	58 (42.0) ^b^		275 (32.7)	144 (28.2) ^a^	58 (29.9) ^a^	73 (52.9) ^b^	
No	642 (76.2)	407 (79.8) ^a^	155 (79.9) ^a^	80 (58.0) ^b^		567 (67.3)	366 (71.8) ^a^	136 (70.1) ^a^	65 (47.1) ^b^	
**Addition of seasonings**					<0.001					<0.001
Yes	386 (45.8)	202 (39.6) ^a^	95 (49.0) ^a^	89 (64.5) ^b^		474 (56.3)	260 (51.0) ^a^	116 (59.8) ^a,b^	98 (71.0) ^b^	
No	456 (54.2)	308 (60.4) ^a^	99 (51.0) ^a^	49 (35.5) ^b^		368 (43.7)	250 (49.0) ^a^	78 (40.2) ^a,b^	40 (29.0) ^b^	
**Blending of food**					0.001					0.031
Yes	420 (49.9)	232 (45.5) ^a^	102 (52.6) ^a,b^	86 (62.3) ^b^		275 (32.7)	167 (32.7) ^a,b^	52 (26.8) ^a^	56 (40.6) ^b^	
No	422 (50.1)	278 (54.5) ^a^	92 (47.4) ^a,b^	52 (37.7) ^b^		567 (67.3)	343 (67.3) ^a,b^	142 (73.2) ^a^	82 (59.4) ^b^	
**Child self-feeding practices**					<0.001					0.035
Yes	662 (78.6)	373 (73.1) ^a^	174 (89.7) ^b^	115 (83.3) ^b^		777 (92.3)	462 (90.6) ^a^	187 (96.4) ^b^	128 (92.8) ^a,b^	
Finger food	652 (77.4)	366 (92.0) ^a^	173 (97.2) ^a^	113 (95.8) ^a^		772 (91.7)	460 (98.5) ^a^	187 (100.0) *	125 (95.4) ^b^	
Cup feed	165 (19.6)	64 (18.3) ^a^	70 (43.2) ^b^	31 (27.4) ^a^		285 (33.8)	116 (25.7) ^a^	108 (58.4) ^b^	61 (47.7) ^b^	
Spoon feed	61 (7.2)	24 (7.0) ^a^	22 (13.8) ^b^	15 (13.2) ^a,b^		115 (13.7)	47 (10.5) ^a^	45 (24.5) ^b^	23 (17.8) ^a,b^	
No	180 (21.4)	137 (26.9) ^a^	20 (10.3) ^b^	23 (16.7) ^b^		65 (7.7)	48 (9.4) ^a^	7 (3.6) ^b^	10 (7.2) ^a,b^	

*p*-value indicates significance level among three ethnic groups (Chinese, Malay, Indian); *p* ≤ 0.05 is considered significant. Values in italics do not add up to the total “Yes” because respondents could have more than one option. ^a,b^ Values in the same row not sharing the same subscript are significantly different at *p* ≤ 0.05 in the two-sided test of equality for column proportions. * This category is not used in comparisons because its column proportion is equal to zero or one.

**Table 4:** Comparison of drinking practices at 9- and 12-month time points among Chinese, Malay and Indian infants (*n* (%)).

	Total Infants at 9 Months	Chinese	Malay	Indian	*p*-Value	Total Infants at 12 Months	Chinese	Malay	Indian	*p*-Value
	(*n* = 842)	(*n* = 510)	(*n* = 194)	(*n* = 138)		(*n* = 842)	(*n* = 510)	(*n* = 194)	(*n* = 138)	
**Drinking of milk**					0.010					0.153
Breastfed	110 (13.1)	64 (12.5) ^a^	21 (10.8) ^a^	25 (18.1) ^a^		90 (10.7)	58 (11.4)	14 (7.2)	18 (13.0)	
Milk Bottle	721 (85.6)	441 (86.5) ^a^	170 (87.6) ^a^	110 (79.7) ^a^		736 (87.4)	442 (86.7)	178 (91.8)	116 (84.1)	
Cup	3 (0.4)	0 (0.0) *	3 (1.5)	0 (0.0) *		5 (0.6)	2 (0.4)	1 (0.5)	2 (1.4)	
Spoon	7 (0.8)	5 (1.0) ^a^	0 (0.0) *	2 (1.4) ^a^		8 (1.0)	7 (1.4)	0 (0.0)	1 (0.7)	
Others	1 (0.1)	0 (0.0) *	0 (0.0) *	1 (0.7)		3 (0.4)	1 (0.2)	1 (0.5)	1 (0.7)	
**Addition of food items into milk bottle**					0.115					0.138
Yes	265 (31.5)	158 (31.0)	54 (27.8)	53 (38.4)		284 (33.7)	172 (33.7)	57 (29.4)	55 (39.9)	
No	577 (68.5)	352 (69.0)	140 (72.2)	85 (61.6)		558 (66.3)	338 (66.3)	137 (70.6)	83 (60.1)	
**Drinking juices and sweetened drinks**					<0.001					<0.001
Milk Bottle	334 (39.7)	186 (36.5) ^a^	80 (41.2) ^a,b^	68 (49.3) ^b^		288 (34.2)	166 (32.5) ^a^	61 (31.4) ^a,b^	61 (44.2) ^b^	
Cup	83 (9.9)	34 (6.7) ^a^	35 (18.0) ^b^	14 (10.1) ^a,b^		137 (16.3)	51 (10.0) ^a^	58 (29.9) ^b^	28 (20.3) ^b^	
Spoon	125 (14.8)	86 (16.9) ^a^	17 (8.8) ^b^	22 (15.9) ^a,b^		108 (12.8)	74 (14.5) ^a^	18 (9.3) ^a^	16 (11.6) ^a^	
Others	34 (4.0)	15 (2.9) ^a^	13 (6.7) ^a^	6 (4.3) ^a^		86 (10.2)	55 (10.8) ^a^	22 (11.3) ^a^	9 (6.5) ^a^	
Do Not Drink	266 (31.6)	189 (37.1) ^a^	49 (25.3) ^b^	28 (20.3) ^b^		223 (26.5)	164 (32.2) ^a^	35 (18.0) ^b^	24 (17.4) ^b^	
**Drinking water**					0.039					<0.001
Milk Bottle	571 (67.8)	350 (68.6) ^a^	125 (64.4) ^a^	96 (69.6) ^a^		459 (54.5)	275 (53.9) ^a^	108 (55.7) ^a^	76 (55.1) ^a^	
Cup	146 (17.3)	73 (14.3) ^a^	45 (23.2) ^b^	28 (20.3) ^a,b^		194 (23.0)	90 (17.6) ^a^	60 (30.9) ^b^	44 (31.9) ^b^	
Spoon	95 (11.3)	68 (13.3) ^a^	16 (8.2) ^a^	11 (8.0) ^a^		64 (7.6)	51 (10.0) ^a^	4 (2.1) ^b^	9 (6.5) ^a,b^	
Others	30 (3.6)	19 (3.7) ^a^	8 (4.1) ^a^	3 (2.2) ^a^		125 (14.8)	94 (18.4) ^a^	22 (11.3) ^a,b^	9 (6.5) ^b^	

*p*-value indicates significance level among three ethnic groups (Chinese, Malay, Indian); *p* ≤ 0.05 is considered significant. ^a,b^ Values in the same row not sharing the same subscript are significantly different at *p* ≤ 0.05 in the two-sided test of equality for column proportions. * This category is not used in comparisons because its column proportion is equal to zero or one.

## Supplementary Materials

The following are available online at http://www.mdpi.com/2072-6643/8/5/293/s1, Figure S1. Flowchart of participants included for analysis in this study, Table S1a. Profile of the mothers in the cohort and supplement intake in their infant (*n* (%)), Table S1b. Profile of the infants in the cohort and supplement intake (*n* (%)).

## Abbreviations

The following abbreviations are used in this manuscript:

GUSTO	Growing Up in Singapore Towards healthy Outcomes
NUH	National University Hospital
KKH	KK Women’s and Children’s Hospital
BMI	Body Mass Index
WHO	World Health Organization
SD	Standard Deviation
BF	Breastfeeding
