# Supplementary material for: Infant Feeding Practices in a Multi-Ethnic Asian Cohort: The GUSTO Study
Source: Nutrients. 2016 May 13;8(5):293. doi: 10.3390/nu8050293 (PMC4882706; doi:10.3390/nu8050293)
Supplement: Supplementary file 1 [file nutrients-08-00293-s001.docx]

Supplementary Materials: Infant Feeding Practices in a Multi-Ethnic Asian Cohort: The GUSTO Study

Jia Ying Toh, Grace Yip, Wee Meng Han, Doris Fok, Yen-Ling Low, Yung Seng Lee, Salome A. Rebello, Seang-Mei Saw, Kenneth Kwek, Keith M. Godfrey, Yap-Seng Chong and Mary Foong-Fong Chong


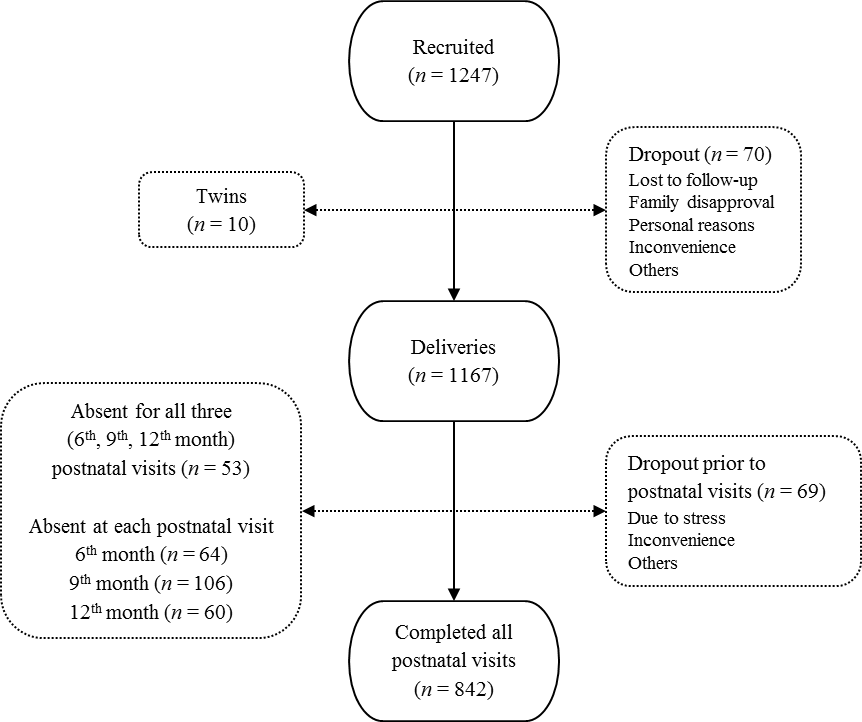


**Figure S1.** Flowchart of participants included for analysis in this study.

**Table S1a.** Profile of the mothers in the cohort and supplement intake in their infant (*n* (%)).

|  | **Infant Consumed Any Supplements?** | | ***p*-Value** |
| --- | --- | --- | --- |
|  | **Yes** | **No** |  |
| Ethnicity |  |  | <0.001 |
| Chinese | 216 (72.2) | 294 (54.1) |  |
| Malay | 35 (11.7) | 159 (29.3) |  |
| Indian | 48 (16.1) | 90 (16.6) |  |
| Age (years) |  |  | <0.001 |
| 18–24 | 18 (6.0) | 61 (11.2) |  |
| 25–29 | 70 (23.4) | 175 (32.2) |  |
| 30–34 | 124 (41.5) | 167 (30.8) |  |
| ≥35 | 87 (29.1) | 140 (25.8) |  |
| BMI (26 weeks) |  |  | 0.846 |
| ≤18.5–22.9 | 70 (23.4) | 118 (21.7) |  |
| 23.0–27.5 | 143 (47.8) | 263 (48.4) |  |
| >27.5 | 86 (28.8) | 162 (29.8) |  |
| Highest education |  |  | <0.001 |
| Secondary or lower | 57 (19.1) | 175 (32.2) |  |
| Post-secondary | 121 (40.5) | 185 (34.1) |  |
| University or higher | 121 (40.5) | 183 (33.7) |  |
| Monthly household income (S$) |  |  | 0.005 |
| ≤1999 | 29 (9.7) | 85 (15.7) |  |
| 2000–5999 | 163 (54.5) | 313 (57.6) |  |
| ≥6000 | 107 (35.8) | 145 (26.7) |  |
| Employment status |  |  | 0.037 |
| Employed | 226 (75.6) | 372 (68.5) |  |
| Unemployed | 73 (24.4) | 171 (31.5) |  |
| Alcohol consumption before pregnancy |  |  | 0.012 |
| Yes | 120 (40.1) | 170 (31.3) |  |
| No | 179 (59.9) | 373 (68.7) |  |
| Smoking before pregnancy |  |  | 0.605 |
| Yes | 32 (10.7) | 66 (12.2) |  |
| No | 267 (89.3) | 477 (87.8) |  |
| Parity |  |  | 0.148 |
| No child | 144 (48.2) | 232 (42.7) |  |
| ≥1 child | 155 (51.8) | 311 (57.3) |  |
| Dietary intake during pregnancy |  |  | 0.946 |
| Vegetarian | 7 (2.3) | 11 (2.0) |  |
| Vegan | 0 (0.0) | 1 (0.2) |  |
| Diabetic diet | 3 (1.0) | 7 (1.3) |  |
| Low fat diet | 6 (2.0) | 9 (1.7) |  |
| Others | 5 (1.7) | 9 (1.7) |  |
| No | 275 (92.0) | 495 (91.2) |  |
| Not reported | 3 (1.0) | 11 (2.0) |  |

Missing information for BMI (*n* = 25), education level (*n* = 7), monthly household income (*n* = 54), employment status (*n* = 16), alcohol consumption (*n* = 12), smoking (*n* = 10) and parity (*n* = 8) were imputed with their respective median values. *p*-value indicates significance level among three ethnic groups (Chinese, Malay, Indian); *p* ≤ 0.05 is considered significant.

**Table S1b.** Profile of the infants in the cohort and supplement intake.

|  | **Infant Consumed Any Supplements?** | | ***p*-Value** |
| --- | --- | --- | --- |
|  | **Yes** | **No** |  |
| Birth weight (kg) (mean (SD)) | 3.08 (0.5) | 3.14 (0.4) | 0.105 |
| Birth length (cm) (mean (SD)) | 48.5 (2.6) | 48.7 (2.1) | 0.258 |
| Gestational age (weeks) (mean (SD)) | 38.2 (1.8) | 38.4 (1.2) | 0.069 |
| Gestational age (weeks) (*n* (%)) |  |  | 0.037 |
| <37 | 28 (9.4) | 29 (5.3) |  |
| ≥37 | 271 (90.6) | 514 (94.7) |  |
| Gender (*n* (%)) |  |  | 0.638 |
| Male | 150 (50.2) | 283 (52.1) |  |
| Female | 149 (49.8) | 260 (47.9) |  |
| Breastfeeding (BF) status (*n* (%)) |  |  | 0.226 |
| Exclusively BF first three months | 53 (17.7) | 111 (20.4) |  |
| Partial BF | 211 (70.6) | 352 (64.8) |  |
| Never BF | 35 (11.7) | 80 (14.7) |  |
| Main food decision maker (*n* (%)) |  |  | 0.026 |
| Mother | 206 (68.9) | 390 (71.8) |  |
| Grandparent | 45 (15.1) | 99 (18.2) |  |
| Others ^1^ | 48 (16.1) | 54 (9.9) |  |
| Age of introduction of first foods (weeks) (*n* (%)) |  |  | 0.688 |
| ≤15 | 8 (2.7) | 12 (2.2) |  |
| 16–23 | 96 (32.1) | 196 (36.1) |  |
| 24–31 | 183 (61.2) | 313 (57.6) |  |
| ≥32 | 12 (4.0) | 22 (4.1) |  |
| First foods (*n* (%)) |  |  | 0.479 |
| Rice cereal | 159 (53.2) | 293 (54.0) |  |
| Non-rice cereal ^2^ | 57 (19.1) | 88 (16.2) |  |
| Rice porridge ^3^ | 31 (10.4) | 71 (13.1) |  |
| Fruits | 23 (7.7) | 36 (6.6) |  |
| Vegetable puree | 11 (3.7) | 20 (3.7) |  |
| Rice | 8 (2.7) | 11 (2.0) |  |
| Baby biscuit | 2 (0.7) | 13 (2.4) |  |
| Others | 5 (1.7) | 9 (1.7) |  |
| Not answered | 3 (1.0) | 2 (0.4) |  |

Missing information for birth length (*n* = 2) and age of introduction of first foods (*n* = 4) was imputed with its median value. *p*-value indicates significance level among three ethnic groups (Chinese, Malay, Indian); *p* ≤ 0.05 is considered significant. ^1^ Others consist of father (*n* = 7), secondary caregiver (*n* = 44), shared responsibility (*n* = 43), not reported (*n* = 8); ^2^ For example, of the non-rice cereal are oats, wholegrain and fruit cereals; ^3^ Plain rice porridge (*n* = 85), flavored rice porridge (*n* = 17).
